# Advanced Knee Structure Analysis (AKSA): a comparison of bone mineral density and trabecular texture measurements using computed tomography and high-resolution peripheral quantitative computed tomography of human knee cadavers

**DOI:** 10.1186/s13075-016-1210-z

**Published:** 2017-01-10

**Authors:** Torsten Lowitz, Oleg Museyko, Valérie Bousson, Christine Chappard, Liess Laouisset, Jean-Denis Laredo, Klaus Engelke

**Affiliations:** 1 Institute of Medical Physics, University of Erlangen-Nürnberg, Henkestr. 91, 91052 Erlangen, Germany; 2 AP-HP, Hôpital Lariboisière, Service de Radiologie Ostéo-Articulaire, 2, rue Ambroise-Paré, F-75475 Paris, Cedex 10, France; 3 Univ. Paris Diderot, Sorbonne Paris Cité, Laboratoire B2OA, CNRS UMR 7052, 75010 Paris, France

**Keywords:** Knee OA, Subchondral bone, Texture, Computed tomography, High-resolution peripheral quantitative computed tomography

## Abstract

**Background:**

A change of loading conditions in the knee causes changes in the subchondral bone and may be a cause of osteoarthritis (OA). However, quantification of trabecular architecture in vivo is difficult due to the limiting spatial resolution of the imaging equipment; one approach is the use of texture parameters. In previous studies, we have used digital models to simulate changes of subchondral bone architecture under OA progression. One major result was that, using computed tomography (CT) images, subchondral bone mineral density (BMD) in combination with anisotropy and global homogeneity could characterize this progression.

The primary goal of this study was a comparison of BMD, entropy, anisotropy, variogram slope, and local and global inhomogeneity measurements between high-resolution peripheral quantitative CT (HR-pQCT) and CT using human cadaveric knees. The secondary goal was the verification of the spatial resolution dependence of texture parameters observed in the earlier simulations, two important prerequisites for the interpretation of in vivo measurements in OA patients.

**Method:**

The applicability of texture analysis to characterize bone architecture in clinical CT examinations was investigated and compared to results obtained from HR-pQCT. Fifty-seven human knee cadavers (OA status unknown) were examined with both imaging modalities. Three-dimensional (3D) segmentation and registration processes, together with automatic positioning of 3D analysis volumes of interest (VOIs), ensured the measurement of BMD and texture parameters at the same anatomical locations in CT and HR-pQCT datasets.

**Results:**

According to the calculation of dice ratios (>0.978), the accuracy of VOI locations between methods was excellent. Entropy, anisotropy, and global inhomogeneity showed significant and high linear correlation between both methods (0.68 < *R* ^2^ < 1.00). The resolution dependence of these parameters simulated earlier was confirmed by the in vitro measurements.

**Conclusion:**

The high correlation of HR-pQCT- and CT-based measurements of entropy, global inhomogeneity, and anisotropy suggests interchangeability between devices regarding the quantification of texture. The agreement of the experimentally determined resolution dependence of global inhomogeneity and anisotropy with earlier simulations is an important milestone towards their use to quantify subchondral bone structure. However, an in vivo study is still required to establish their clinical relevance.

## Background

The assessment of trabecular structure of subchondral bone has become an important research area in osteoarthritis (OA) [1–6]. In particular, the association between early OA and altered loading conditions causing remodeling of the fine trabecular network has received recent attention [7–9]. However, quantification of trabecular structure in vivo is difficult. Typically, a high-spatial resolution computed tomography (CT) dataset is binarized to segment the trabecular network, which then can be quantified using standard histomorphometric parameters such as trabecular separation, thickness, or number. However, microcomputed tomography (μCT), the current gold-standard for the three-dimensional (3D) quantification of trabecular structure, is not applicable in humans in vivo. High-resolution peripheral quantitative computed tomography (HR-pQCT) imaging with a spatial resolution of about 120 μm [10] is limited to distal locations such as fingers, the distal radius, or the distal tibia. The knee or hip, which are important locations for OA, cannot be assessed. Also, scan times are long, often resulting in motion artifacts that prevent an accurate analysis of the trabecular network. Imaging techniques such as CT and magnetic resonance imaging (MRI), which use clinical whole body scanners, still do not offer the spatial resolution necessary to segment trabeculae.

Recently, we have addressed this problem with a grey-level texture analysis applied to the subchondral bone of the knee [11] which does not require a segmentation of the trabecular network. Texture describes the distribution of grey values. In contrast, bone mineral density (BMD), after appropriate calibration, is a mean of grey values. The result of a texture measurement depends on image noise and spatial resolution. Therefore, the interpretation of such measurements, for example from CT images of the knee, is not straightforward and the impact of disease or progression of disease on texture measurements is largely unknown which limits their clinical applicability. In two recent papers, we have used digital bone models to better understand texture by simulating a variety of trabecular bone structures and the imaging process at different spatial resolutions from μCT (20 μm), HR-pQCT (120 μm), and clinical whole-body CT (400 μm) scanners [12]. We specifically simulated changes in subchondral trabecular bone structure with OA [13] and investigated which combination of texture parameters may be best suited to quantify these changes at different spatial resolutions. We showed that BMD alone cannot be used for this purpose, but BMD in combination with global inhomogeneity and anisotropy might be applicable even when patients are investigated with clinical whole-body CT scanners. A detailed description was given in [11] and [12].

The current cadaver study extends these prior investigations. Here, subchondral bone texture of real bones is investigated at voxel sizes (HR-pQCT and CT) simulated earlier. It was not our aim to investigate OA versus non-OA knees or the impact of OA progression on bone texture; the task was to demonstrate clinical relevance of quantifying bone texture. Specifically, the primary goal was to compare texture measurements characterizing trabecular bone structure between HR-pQCT and whole-body quantitative CT (QCT) using human cadaveric knees. The secondary goal was the verification of the spatial resolution dependence observed in the earlier simulations [12, 13]. To our knowledge, a comparison of texture parameters measured at different spatial resolutions in the knee has not been reported.

The study reported here is another step towards our ultimate goal to quantify the characteristics of subchondral bone density and architecture and to use these parameters to determine progression or to monitor treatment of OA in the knee. As shown in our previous studies, the use of texture parameters is promising but their relevance when applied in vivo is difficult to understand. Therefore, the current study is important to validate the previously simulated dependence of texture parameters on spatial resolution, a prerequisite for comparison of OA patients and normal controls.

## Methods

### Patients

Fifty-seven cadaveric human knees from 32 subjects (18 females, 83 ± 8 years; 14 males, 79 ± 11 years) were included in the study. Whole knee cadavers were scanned in order to approach the in vivo situation as closely as possible. The cadavers were obtained from the Saint-Pères Pathology Laboratory, Paris VI, France, from subjects who had bequeathed their bodies to science. Further information on the subjects was not available. The study was approved by the ethics committee of Descartes University, Paris. The whole knees, including soft tissues, were harvested in compliance with institutional safety regulations and were kept at –20 °C.

### Image acquisition

**Fig. 1:**
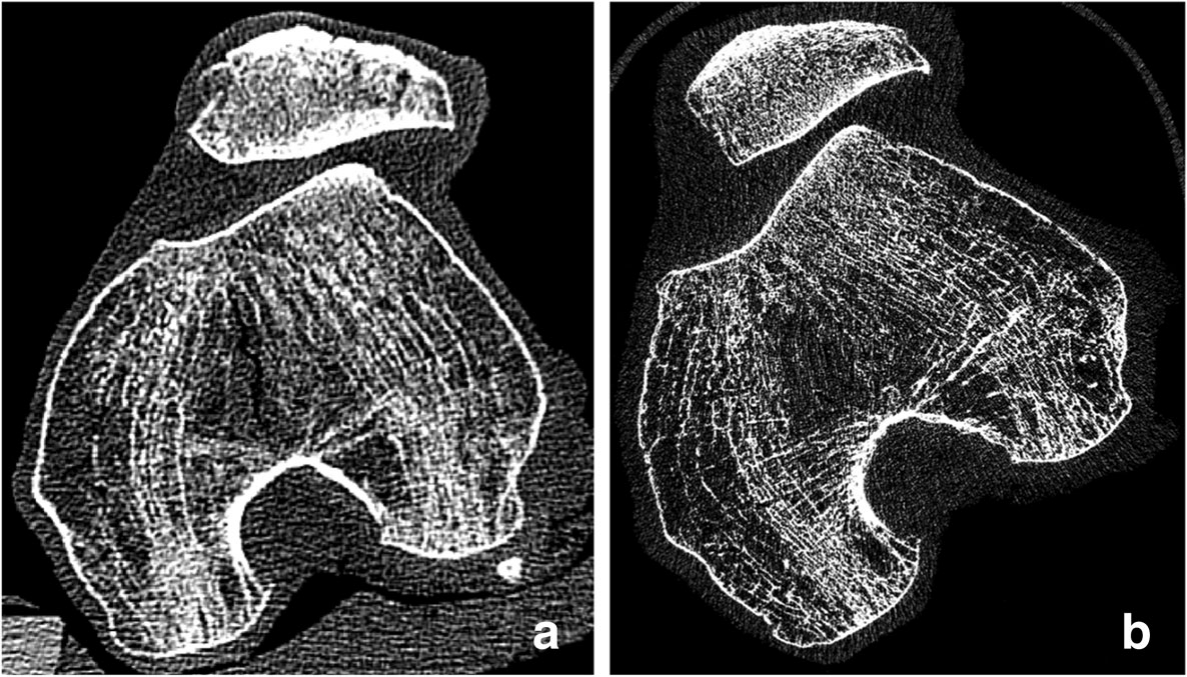
Axial slice of one specimen obtained from clinical CT using a high-resolution kernel (**a**) and from HR-pQCT (**b**)

QCT as well as HR-pQCT data were obtained from all knees (see example in Fig. 1). All QCT datasets were acquired on a Siemens Sensation 64 scanner using the following protocol: 120 kV, 200 mAs, slice thickness 0.5 mm, reconstruction increment 0.3 mm, field of view 13 cm (corresponding to an in-plane pixel size of 250 μm), and a scan length of 20 cm. The CT data were reconstructed with a medium reconstruction (U40u) and a sharp reconstruction kernel (U70u). Datasets reconstructed with the U40u kernel were used for segmentation and BMD analysis. Datasets reconstructed with the U70u kernel were used for texture analysis. An in-scan calibration phantom (Siemens OSTEO phantom) using a mixture of CaCO_3_ and MgO to represent bone [14] was placed under the knees during the image acquisition in order to convert the measured CT values to BMD. Central quality control of all CT examinations was performed by the same radiologist (LL).

HR-pQCT data were acquired on an XtremeCT scanner (Scanco Medical AG, Switzerland) using the following protocol: 59.4 kV, 90 μAs, isotropic voxels with an edge length of 82 μm, and scan length 6–8 cm. An internal calibration based on phantom scans acquired separately from the cadaver scans allowed the automatic conversion of CT values to BMD. The phantom used by Scanco contains hydroxyapatite to represent bone. All HR-pQCT examinations were performed by the same technician. As different phantoms consisting of slightly different materials are used for the BMD calibration, the BMD values in the CT and HR-pQCT datasets also differ.

### Image analysis (segmentation and registration)

Image analysis was performed using MIAF-Knee software (MIAF: Medical Image Analysis Framework), as described in detail previously [11]. In brief, periosteal/articular bone surfaces of the distal femur and the proximal tibia were segmented separately in the CT and HR-pQCT datasets. Then, in the CT datasets, the shaft axes and planar approximations of the growth plates were used for an automatic definition of analysis volumes of interest (VOIs). In order to ensure that the BMD and texture analysis was performed exactly in the same anatomical location, the periosteal/articular surface was registered rigidly from the CT dataset to the corresponding HR-pQCT dataset. The resulting transformation matrix was used to transfer the analysis VOIs from the CT to the HR-pQCT dataset. The Insight Segmentation and Registration Toolkit (ITK) library [15] was used for the registration processes.

To check for registration accuracy, dice ratios [16] between segmented and registered periosteal surfaces were calculated in HR-pQCT datasets to quantify the overlap between both volumes after the registration process. CT datasets were upsized. Dice ratios were determined separately for the femur and tibia. A dice ratio of 1 indicates perfect overlap.

### Image analysis (BMD and texture measurements)

**Fig. 2:**
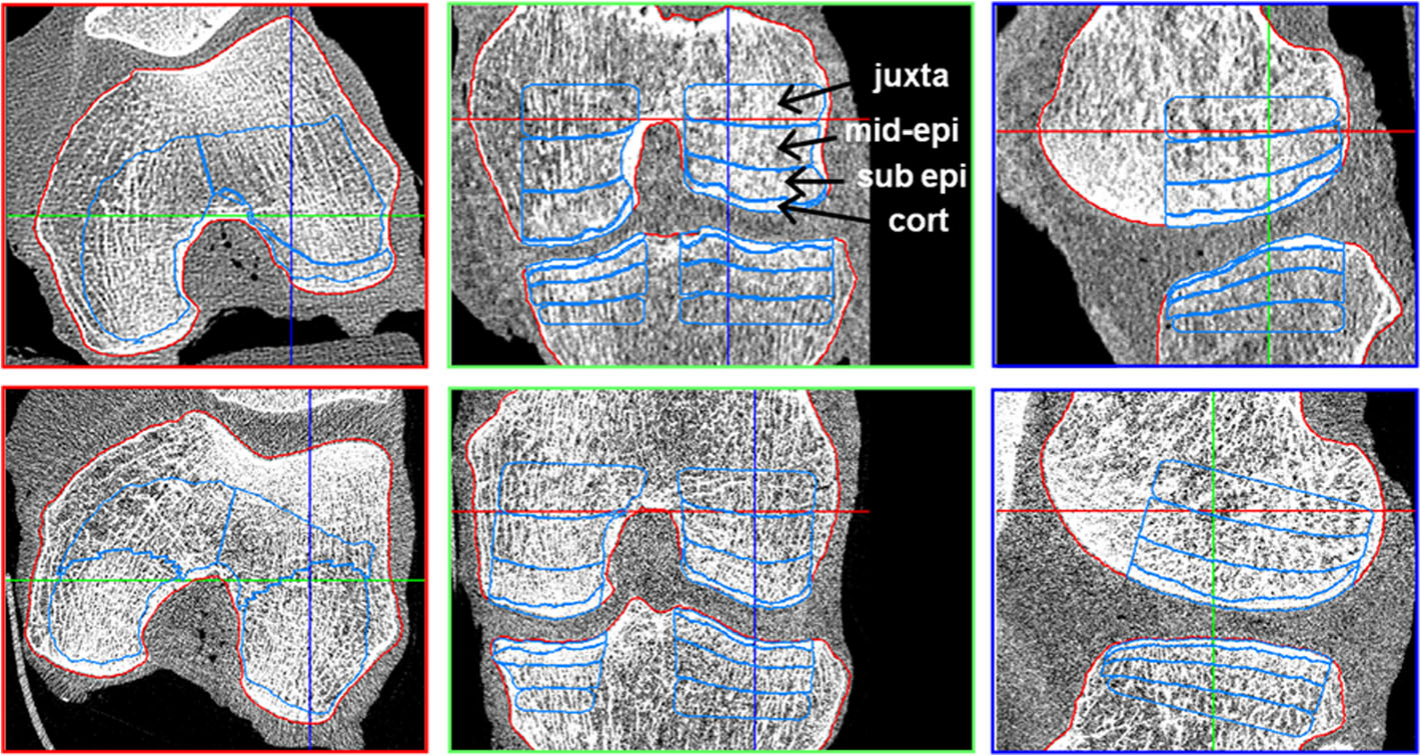
Multi-planar reformations: transversal (*left*), coronal (*center*) and sagittal (right). *Top* CT dataset with segmented periosteal/articular surface (*red*) and analysis VOIs (*blue*); for the CT reconstruction, the high-resolution kernel U70u was applied. *Bottom* HR-pQCT dataset of the same knee (repositioned) with periosteal/articular surface registered (*red*) and analysis VOIs (*blue*) transferred from the CT dataset. The names of the analyses VOIs are only indicated in the femur (*top*, *center*) but apply to the tibia as well. For the purpose of illustration, the HR-pQCT was downsampled to the same size as the CT dataset. Each CT image has 512 × 512 pixels with a size of 254 × 254 μm^2^ each, while the HR-pQCT image consists of 1352 × 1484 pixels with a size of 82 × 82 μm^2^. Navigation lines were added to every image in order to indicate the relative positions of the reformed slices. *cort* cortical, *mid-epi* mid-epiphyseal, *sub epi* subchondral epiphyseal

The main analysis VOIs in the tibia and femur were cortical, subchondral epiphyseal, mid-epiphyseal, and juxta-physeal VOIs (Fig. 2) [11]. In each of them, BMD and texture analyses were performed separately for the medial and lateral compartments. With this approach a total of 16 VOIs were used. Five texture parameters were measured [12]: entropy, global inhomogeneity, local inhomogeneity, anisotropy, and variogram slope. Texture values depend on grey values; thus, for the comparison between CT and HR-pQCT in this study, texture parameters were calculated after calibrating to BMD values [12, 13]. These parameters were selected based on their monotonic response to changes of OA-related structure modifications across different spatial resolutions [12, 13]. In brief, entropy measures information content. Global and local inhomogeneity, which are identical to the standard deviation, measure grey value fluctuations on a global (VOI) or local neighborhood scale. Local anisotropy represents the variation of directedness in a local neighborhood, and variogram slope, which is also the basis of the trabecular bone score, describes mean grey value difference between voxels at a given distance.

### Statistical analysis

For each analysis parameter and VOI, mean values from all 57 knees were calculated separately for CT and HR-pQCT datasets. For 26 pairs of right and left cadavers from the same subject, results were averaged before further analyses. Differences between the two modalities were investigated by linear regression analysis and Bland-Altman plots [17]. The regression results were used to correct the systematic difference in BMD results between CT and HR-pQCT datasets caused by differences in the calibration procedure as described in the methods section.

Finally, for each texture parameter, resolution dependence *D* between HR-pQCT and CT analysis results was calculated as:

$$ D=\frac{T{P}_{HR- pQCT}}{T{P}_{CT}} $$

where *TP* denotes one of the five texture parameters. For each cadaver, 12 different *D* values were obtained, one for each VOI (except for the cortical ones). In our earlier study using the digital bone model [12] we had determined the same texture parameters as above for 40 different simulated trabecular structures using spatial resolutions corresponding to HR-pQCT and CT scanners. For each of the 40 digital models, the parameter *D* was also calculated. For this study, mean *D* values calculated as averages from the 40 digital models were compared with mean D values averaged over all 12 values per cadaver and then over all cadavers.

A two-sample Student’s *t* test was performed to detect differences between both methods (digital bone model vs cadaveric datasets). The Shapiro-Wilk and Levene’s tests were used to check for normal distributions and homogeneous variances. For all statistical tests, a *p* value of less than 0.05 was considered statistically significant. IBM® SPSS STATISTICS version 21.0.0.0 was used for all statistical analyses.

## Results

Figure 2 shows a CT dataset with the periosteal/articular segmentation and VOIs as well as the HR-pQCT dataset of the same specimen with the results of the rigid registration of the periosteal/articular surface and the transferred analysis VOIs from the CT dataset.

The independent segmentation of the periosteal/articular surfaces resulted in almost identical surfaces for CT and HR-pQCT, and registration results were excellent. This was confirmed by very high dice ratios for the femur (0.979 ± 0.005, mean ± standard deviation) and tibia (0.978 ± 0.005). When registered to the HR-pQCT datasets, the periosteal/articular surfaces of the CT datasets included some non-bone voxels at the joint space margin. This is a result of the lower spatial resolution in CT causing partial volume artifacts, which artificially extends the appearance of the bone surface. As such, the largest effect was seen in the cortical VOIs.

**Fig. 3:**
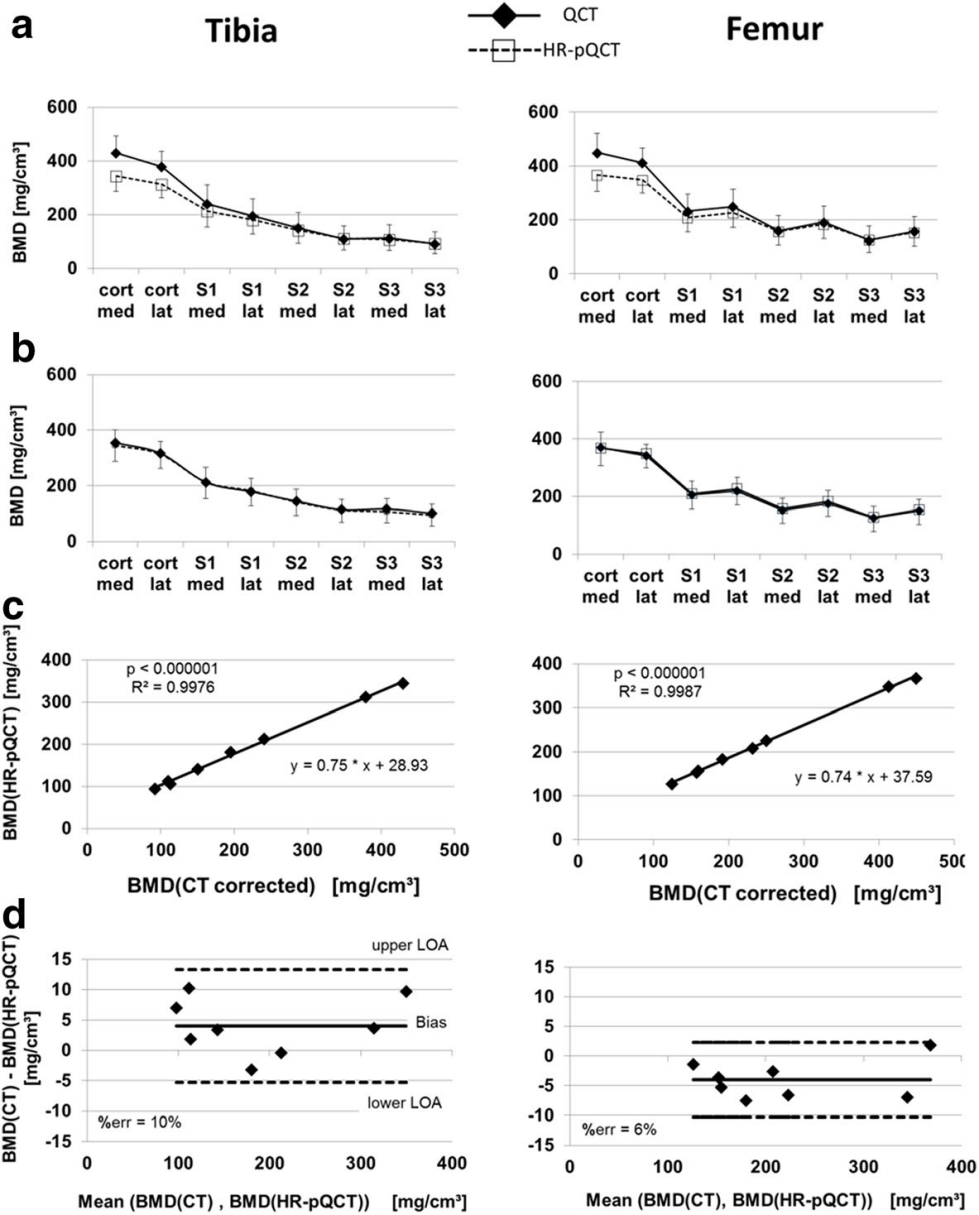
**a** Measured BMD across VOIs for CT and HR-pQCT in tibia and femur with error bars as standard deviations from 57 cadavers. **b** HR-pQCT results unchanged, CT results corrected by the equation obtained from linear regression in (**c**). **c** Linear regression analysis of BMD results. **d** Bland-Altman plots for corrected trabecular BMD. Upper (*lower*) LOA: 95% upper (*lower*) confidence limit (LOA = 1.96 × standard deviation of difference). %err = LOA divided by the mean BMD_HR-pQCT_. *med* medial, *lat* lateral, *LOA* limit of agreement, *S1* subchondral epiphyseal, *S2* mid-epiphyseal, *S3* juxtaphyseal

BMD results between CT and HR-pQCT are compared in Fig. 3. As expected, BMD was highest in the cortical VOIs and decreased with increasing distance from the joint space. Without the correction of the systematic calibration differences, cortical BMD_HR-pQCT_ was on average 18% lower than cortical BMD_CT_ and trabecular BMD_HR-pQCT_ was on average 4% lower than trabecular BMD_CT_ (Fig. 3a). However, BMD_HR-pQCT_ and BMD_CT_ were very highly correlated (*p* < 0.001, *R* ^2^ > 0.997; Fig. 3c). For correction, the linear regression (slope 0.75, intercept 32.0) of the combined tibia and femur results was used.

After correcting BMD_CT_, cortical BMD_CT_ remained 2.0% higher than cortical BMD_HR-pQCT_ in the tibia and 0.7% lower in the femur. Trabecular BMD remained 2.9% higher in the tibia and 2.6% lower in the femur as shown in the Bland-Altman plots (Fig. 3d). The difference did not depend on absolute BMD values. There were no statistical outliers, as all data points were within the limits of agreement and all parameters were normally distributed. As the cortical BMD values were not used for the calibration correction they were also not included in the Bland-Altman plots.

**Fig. 4:**
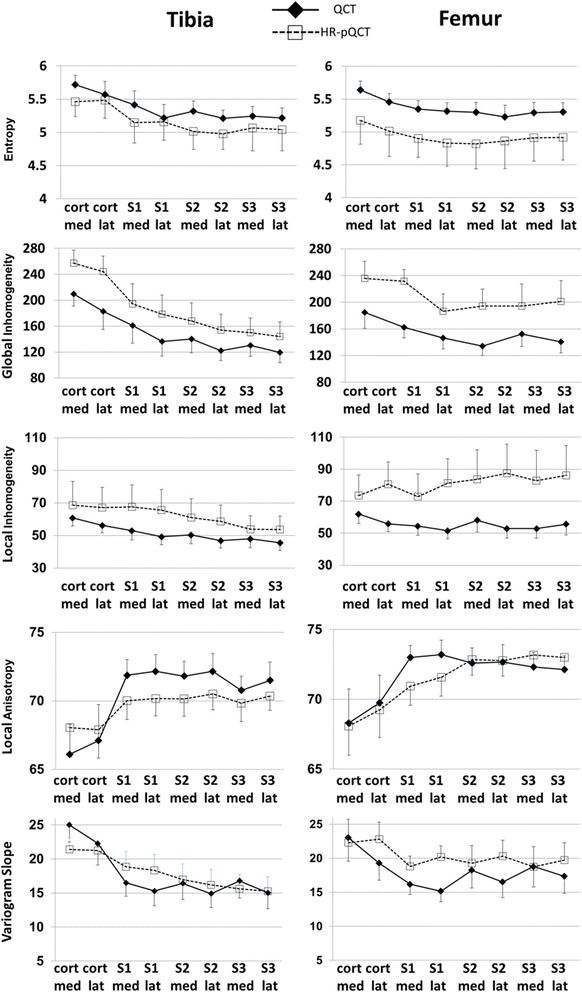
Texture parameters measured with CT or HR-pQCT in the VOIs shown in Fig. 2

**Fig. 5:**
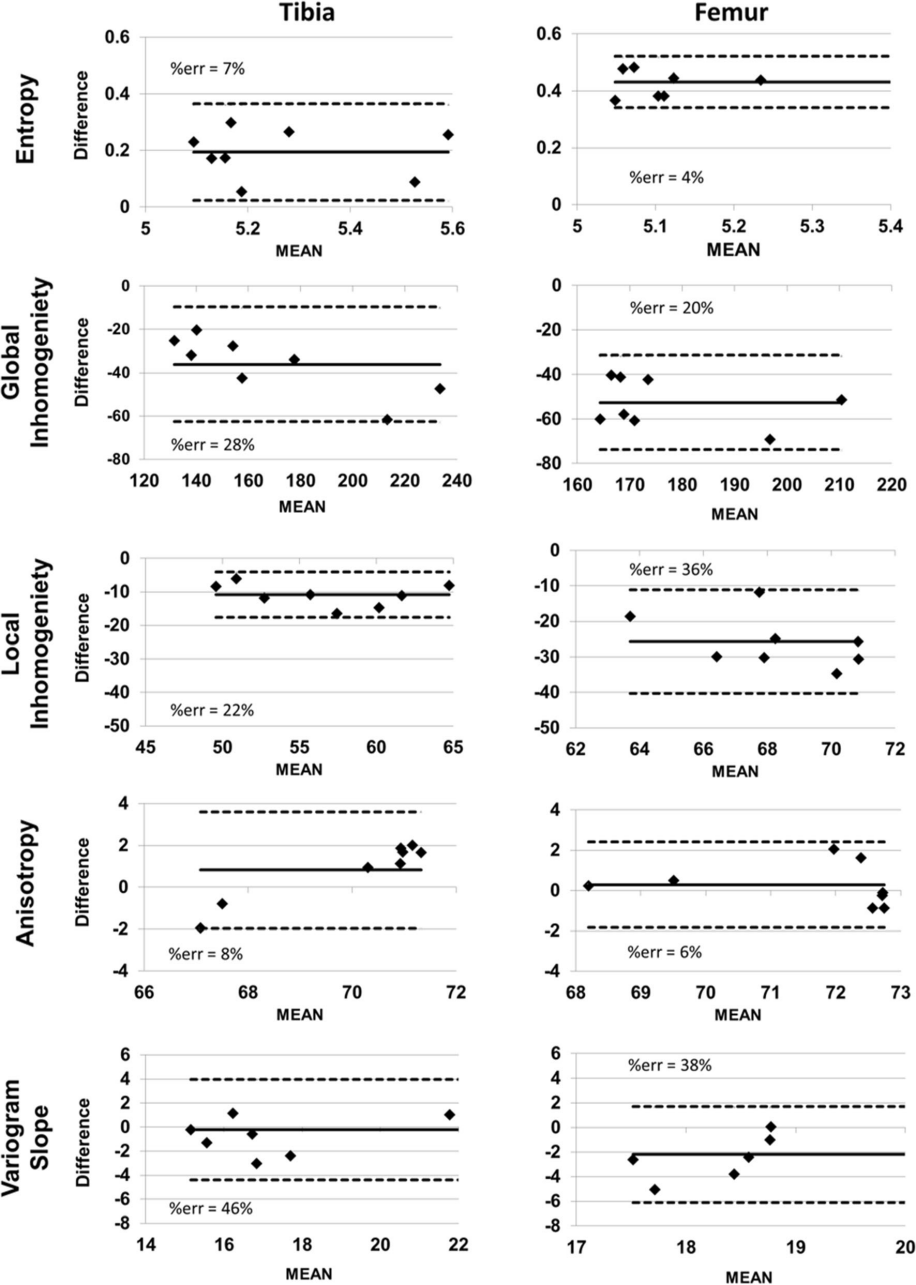
Comparison (using Bland-Altman plots) of texture parameters measured with CT and HR-pQCT. *MEAN* mean of CT and HR-pQCT measurements, *DIFFERENCE* CT measurement in CT – HR-pQCT measurement

Texture results are shown in Fig. 4. *R* ^2^ values and *p* values of the corresponding linear regression analyses are listed in Table 1. With the exception of local inhomogeneity and variogram slope in the femur, all texture parameters showed significant linear correlations between CT and HR-pQCT, with high *R* ^2^ values (≥0.7) in both bones. With the exception of entropy, correlations were higher in the tibia compared to the femur. Texture parameters showed mostly comparable behavior between CT and HR-pQCT. Differences in absolute values between the two modalities were lowest for anisotropy. Bland-Altman plots are shown in Fig. 5. Only anisotropy showed practically no systematic bias. Entropy was higher with CT, whereas variogram slope and global and local inhomogeneity were higher in HR-pQCT datasets. The error was particularly low for entropy and anisotropy. There were no statistical outliers.

**Table 1:** Texture analysis

	Tibia	Femur
Bone mineral density	1.00 (<0.001) [1.0, 3.64]	1.00 (<0.001) [0.99, 5.10]
Entropy	0.79 (0.002) [0.93, 0.16]	0.89 (0.001) [0.86, 0.24]
Global inhomogeneity	0.96 (<0.001) [1.31, –11.0]	0.68 (0.012) [0.98, 56.0]
Local inhomogeneity	0.67 (0.012) [0.96, 13.0]	0.22 (0.265) [–0.72, 121]^a^
Anisotropy	0.96 (<0.001) [0.43, 39.3]	0.70 (0.011) [0.93, 4.66]
Variogram slope	0.72 (0.008) [0.54, 8.37]	0.34 (0.136) [0.37, 13.6]^a^

Results are shown as *R* ^2^ values (*p* values) [slope, intercept] of linear regression analyses between CT and HR-pQCT results

^a^Non-significant linear regressions

**Fig. 6:**
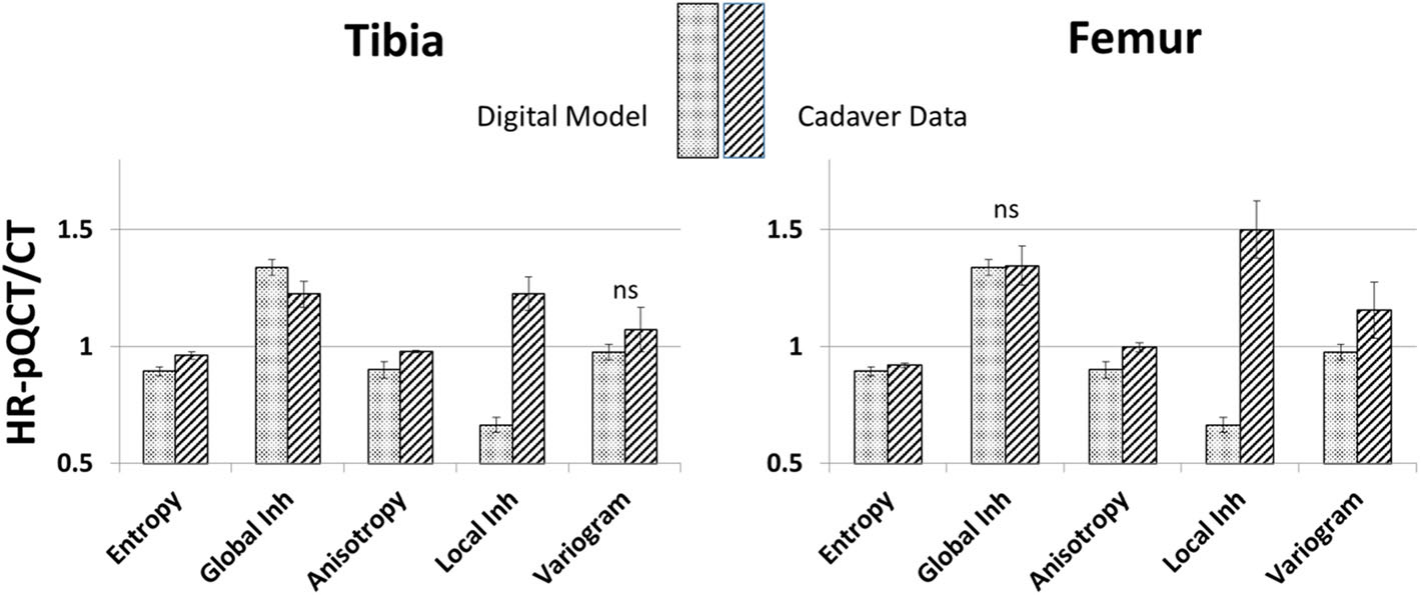
Texture parameter ratios *D* between HR-pQCT and CT measurements. Bars are mean values for 40 digital models simulating a wide variety of trabecular architectures and mean values from twelve trabecular VOIs of 57 cadaveric datasets, respectively. Error bars represent the respective standard deviations. A value of 1 means that the texture parameter does not depend on spatial resolution within the investigated range from about 100 μm (HR-pQCT) to 400 μm (CT)

With respect to the second goal, texture parameter ratios *D* between HR-pQCT and CT datasets are shown in Fig. 6. In the tibia, differences between data from the digital model and the ex vivo datasets were below 10% for entropy and global inhomogeneity, and below 20% for anisotropy and variogram slope. In the femur, differences were below 10% for entropy, global inhomogeneity, anisotropy, and variogram slope. Differences for local inhomogeneity were considerably higher in the tibia (85%) and femur (125%). All differences were significant with the exception of variogram slope in the tibia and global inhomogeneity in the femur.

## Discussion

The in vivo assessment of trabecular structure is a recurring topic to complement BMD measurements in osteoporosis [18–22] or to assess changes in subchondral trabecular bone structure, which may be associated with early OA [23–25]. However, the interpretation of bone texture remains challenging. For example, anisotropy describes the directedness of trabecular structure, but changes in anisotropy with increasing severity of OA depend on assumptions about how OA modifies the trabecular architecture and on spatial resolution [13]. Thus, the clinical meaning of an anisotropy measurement is not immediately obvious. Regarding other texture parameters, such as entropy or variogram slope, it is already difficult to understand which structural component of the network they characterize. The dependence of texture on spatial resolution and noise significantly adds to difficulties in their interpretation. Finally, there are a large variety of texture parameters and there is no clear strategy which to pick for a given clinical question.

In order to improve the interpretability of texture parameters, we previously [12] developed a digital bone model to simulate different architectures of the trabecular network and the impact of noise and spatial resolution with which texture measurements can be systematically characterized. In a follow-up study [13], we applied this framework to modifications of subchondral bone structure with progressive OA described in the literature [26–33]. We showed that a combination of BMD, global inhomogeneity, and anisotropy could be used to quantify OA-related structural changes in the human trabecular bone network of the knee, even at spatial resolutions achievable with clinical CT equipment. An isolated BMD measurement failed to differentiate these structural changes.

The current study of cadaveric knees confirms the resolution dependence of the texture parameters that was observed in the simulations. This is an important step towards the quantification of trabecular bone structure in vivo with CT imaging. It is a limitation of this study that the OA status of the cadavers was unknown, so we could not verify the results of the simulations with respect to OA progression. However, the results here support the use of anisotropy and global inhomogeneity that were identified as the most important texture parameters in simulations of OA progression. Final in vivo validation in subjects with OA is still required. Nevertheless, the current study is an important milestone towards understanding the clinical relevance of texture parameters because results were obtained from two imaging modalities included in the prior simulations.

Texture parameters as well as BMD were calculated at the same anatomical locations of cadaveric knees in CT and HR-pQCT datasets. As expected, BMD correlated extremely well between the two methods. Density measurements are average values from all voxels of the analyzed VOI and, therefore, typically depend less on spatial resolution and image noise than structure or texture parameters. After the correction for calibration differences, a small BMD-independent bias of no more than 5 mg/cm^3^ remained between the two methods, with slightly higher values in the cortical VOIs (Fig. 3b) which were probably caused by the slightly larger cortical volume obtained in the CT datasets versus the HR-pQCT datasets.

With the exception of local inhomogeneity and variogram slope in the femur, texture results correlated highly between CT and HR-pQCT measurements (Table 1), although biases of up to 47% for variogram slope of the tibia between the two measurements were observed (Fig. 5). Correlations were higher in the tibia than in the femur, with the exception of entropy where they were about equal. This indicates that the tibia is the preferred location in the knee to measure texture, although a constant bias can be considered in the analysis and corrected for if necessary. Thus, even the relatively high differences between CT and HR-pQCT results do not reduce the value of a texture analysis. A consistent progression of texture parameters with changing trabecular structure is far more important than absolute values, thus the regression results in Table 1 deserve more attention than the biases. The differences in texture between CT and HR-pQCT are caused by two effects: higher noise and higher spatial resolution in the HR-pQCT datasets. In general, an increase in noise results in an increase in entropy, global and local inhomogeneity, and variogram slope because the grey value distribution within the analysis VOIs becomes more random. In contrast, anisotropy is largely independent of noise, as shown previously [12]. In the protocols used in the present study, noise was about five times higher with HR-pQCT than with CT.

Independent of noise, the decrease in spatial resolution in CT compared to HR-pQCT changed the grey-value distribution. Due to partial volume artifacts, contrast differences were no longer measured between voxels with a volume of 250 μm^3^ but between voxels with a volume of 82 μm^3^, which considerably smoothed the grey value distribution of the analysis VOI. This is important for the entropy calculation, which is based on the histogram of the grey-value distribution. Entropy was higher in the CT images due to the more uniform distribution in CT, and this effect was stronger than the increased noise observed in HR-pQCT, which also increases entropy [12]. In contrast, global inhomogeneity and variogram slope were higher for HR-pQCT. Here, both effects (higher noise in HR-pQCT and smaller grey-value variations in CT) were additive.

As shown earlier, local inhomogeneity is more sensitive to noise than the other texture parameters included in the analysis [12]. This effect is most likely the main reason for the higher local inhomogeneity in HR-pQCT. The effect of spatial resolution is twofold. The smoother histogram decreases local inhomogeneity. However, in terms of numbers of voxels, homogeneous regions are smaller in CT than in higher resolution HR-pQCT, which increases local inhomogeneity in CT. Thus, the resolution-dependent effects on local inhomogeneity may have been canceled out, leaving noise dependence the main factor causing larger values in HR-pQCT.

In contrast to local inhomogeneity, anisotropy differences between CT and HR-pQCT were almost exclusively caused by differences in spatial resolution, which were driven by two opposing effects. First, as already explained, the increased voxel size in CT caused a decrease in the size of homogeneous regions as measured in number of voxels and therefore led to increasing anisotropy. Second, the simultaneous decrease of grey-value gradients at transitions between bone and soft tissue led to decreasing anisotropy. Here, the former effect is a little more dominant than the second one. According to the results in [12], anisotropy was expected to be slightly higher in CT datasets compared to HR-pQCT datasets, which was mostly confirmed here. However, in the femur differences were low.

The results of this study confirmed earlier simulations of the impact of spatial resolution between HR-pQCT and CT reasonably well. With the exception of local inhomogeneity, the CT and HR-pQCT ratios shown in Fig. 6 were quite similar. Variogram slope of the tibia and global inhomogeneity of the femur showed no differences between simulations and cadaver measurements. This confirmed the applicability of the digital bone model to predict the behavior of texture parameters in a wide range of different realistic scenarios and imaging characteristics. The high discrepancy in local inhomogeneity was mainly caused by a lower than realistic assumed noise level in the digital bone model for HR-pQCT datasets in combination with the rather high noise sensitivity of local inhomogeneity.

Comparing resolution and noise effects using the 40 digital models with those of the scanned cadavers has limitations. The 40 different models represent a large variety of trabecular architectures covering ‘healthy subjects to subjects with severe OA’. In contrast, here the OA status of the cadavers is unknown. However, in an elderly population the prevalence of knee OA is typically high. Despite this uncertainty and the different approaches to calculate means for the resolution dependence *D*, standard deviations shown in Fig. 6 were similar or even higher for the cadaveric data indicating that the variation in texture in the cadavers was at least as high as in the simulated data.

The study had several limitations. First, as already discussed above, there was no information on the OA status of the cadavers. Second, μCT images were not obtained. However, most μCT scanners do not offer a sufficiently large field of view to scan a complete human knee and a μCT study on bone core was beyond the scope of this study. Third, the first generation HR-pQCT equipment used in this study can be used for in vitro but not in vivo scans of knees; therefore, for the purpose of this study we were restricted to a cadaver study. In vivo knee scans have been reported with the second-generation HR-pQCT equipment [34] but will be limited to younger people who can still bend one leg while the other remains stretched. Fourth, only five texture parameters were included in the study, although many more exist. The five parameters used here had been selected earlier based on their monotonic response to changes of OA-related structure modifications across different spatial resolutions.

## Conclusions

After appropriate corrections to account for differences in the calibration phantoms, BMD differences between HR-pQCT and CT were below 3%. Entropy, global inhomogeneity, and anisotropy showed significant and high correlations between both methods (*R* ^2^ > 0.7), suggesting interchangeability between devices regarding the quantification of texture. Results from a previous simulation suggested that the combination of BMD, global inhomogeneity, and anisotropy could be used to characterize changes in subchondral bone architecture with OA progression. In this study, the resolution dependence of global inhomogeneity and anisotropy was confirmed. Future research will evaluate the clinical relevance of these two texture parameters for the detection of early OA in vivo in CT images of the knee.

## Abbreviations

3D: Three-dimensional
AKSA: Advanced Knee Structure Analysis
BMD: Bone mineral density
CT: Computed tomography
HR-pQCT: High-resolution peripheral quantitative computed tomography
OA: Osteoarthritis
QCT: Quantitative computed tomography
VOI: Volume of interest
μCT: Microcomputed tomography
